# Nutritional value and safety of animal feed supplemented with *Talaromyces verruculosus*-treated cocoa pod husks

**DOI:** 10.1038/s41598-020-69763-9

**Published:** 2020-08-04

**Authors:** Daniel Oduro-Mensah, Augustine Ocloo, Thomas Nortey, Stephen Antwi, Laud K. Okine, Naa A. Adamafio

**Affiliations:** 1 Department of Biochemistry, Cell and Molecular Biology, College of Basic and Applied Sciences, University of Ghana, Legon, P. O. Box LG54, Accra, Ghana; 2 Department of Animal Science, College of Basic and Applied Sciences, University of Ghana, Legon, P. O. Box LG226, Accra, Ghana; 3 Department of Pharmacology, Centre for Plant Medicine Research, P. O. Box 73, Mampong-Akwapim, Ghana; 4 Department of Applied Chemistry and Biochemistry, Faculty of Applied Sciences, University for Development Studies, Navrongo Campus, P. O. Box 24, Navrongo, Ghana

**Keywords:** Biochemistry, Animal physiology

## Abstract

Theobromine exerts deleterious effects on animal physiology. Removal of theobromine from the millions of metric tons of cocoa pod husks (CPH) discarded annually could allow for the production of cheap, CPH-based animal feed. The aim of this study was to evaluate safety and nutritional value of bio-detheobrominated CPH in Sprague–Dawley rats. Theobromine was removed from CPH by treatment with an isolate of *Talaromyces verruculosus* (TvTD). Substituted feeds containing CPH were formulated by replacing 30% or 50% of the maize content of regular rat feed with TvTD-treated or inactivated TvTD-treated CPH. Feeding groups included control groups without or with theobromine administration. Effects of the feed formulations on water and feed intake, weight gain, blood biochemistry and organ-specific toxicity were assessed. Rats ingesting theobromine in inactivated TvTD-treated CPH-based diet or by oral gavage variably exhibited marked deleterious effects, mainly evident in body weight, thymus wet weight and tissue histology. In contrast, substitution with TvTD-treated CPH caused significant increase in body weight. Substitution at 30% did not cause mortality or organ-specific toxicity with reference to the testes, kidneys, spleen or liver, unlike substitution at 50%. The data demonstrate that detheobrominated CPH may safely replace up to 30% of maize in animal feed formulations.

## Introduction

In most developing countries, the average diet is low in animal protein^1,2^. Part of the problem is availability and/or price of poultry and livestock meat due to high production costs. It has been suggested that in Ghana, for example, soaring prices of poultry feed were the biggest cost factor limiting production and increasing poultry prices on the market^3^. This underscores the need for work toward the use of agro wastes in animal feed compositions for poultry and livestock.

The use of cocoa pod husks (CPH) as animal feed is a promising application under investigation^4–6^. Whereas inclusion of CPH at 6.5% in broiler diet was found to enhance growth, inclusion at 19.5% depressed performance^7^. Though feed conversion efficiency and net protein utilization were adversely affected, farmed tilapia were found to accept CPH substitution of feed at up to 20% in their diets, as indicated by increased feed consumption and weight gain^8^. In addition to its high fiber content, higher inclusion levels and widespread use of CPH in animal feed has been limited by a number of its constituent compounds, principally theobromine, which has shown detrimental effects such as delayed growth, atrophy of organs and lethality in experimental animals including rats^9–12^. Appropriate treatment to completely remove or reduce the theobromine content could be a big step toward solving the problems currently limiting use of CPH in animal feed.

The application of microorganisms as bio-tools for theobromine removal has been shown to be plausible for use on CPH^13,14^. In related studies, an isolate of *Talaromyces verruculosus* (TvTD) has emerged as a promising bio-tool for use in detheobromination of CPH^15,16^. The current study was aimed at determining the nutritional value and safety of rat feed substituted at different levels with TvTD-treated CPH.

## Materials and methods

### Handling of experimental animals

Animal experiments for this study were conducted at the Animal Experimentation Unit of Centre for Plant Medicine Research (CPMR), Mampong-Akwapim, Ghana using outbred Sprague–Dawley rats purchased from the same Institution. The study received clearance from the Ethical Clearance Committee of CPMR through the Animal Experimentation Unit. All experimental protocols and procedures were approved by the Ethical Clearance Committee of CPMR and were performed according to standard operating procedures and guidelines of the Unit. Throughout the period of experimentation, the animals were handled in accordance with internationally accepted standards and principles of laboratory animal use and care (EEC Directive 2010/63/EU)^17^. During a 2-week acclimatization period, sterilized drinking water and regular rat chow, used as control diet in this study, were provided ad libitum. Formulation of the control diet (Table 1) was based on National Research Council, USA recommendations for rats and mice^18^.

**Table 1:** Formulation and nutrient content of experimental diets.

Item	Control diet (0)	Substitution level 1 (30%)^a^	Substitution level 2 (50%)^a^
**Feed ingredient, kg/100 kg**			
CPH	0	30	50
Maize	60	28.3	3.1
Wheat bran	15.45	10.25	10.5
Soybean meal	22.5	26.5	29
Lysine	0.15	0.05	0
Oil (soybean)	0	3	5.5
Calcium hydrogen phosphate	0.15	0.15	0.15
Limestone	1	1	1
Sodium chloride	0.5	0.5	0.5
Vitamin and mineral premix	0.25	0.25	0.25
**Calculated nutrient content, % DM**			
Crude protein	17.812	17.517	17.524
Fiber	3.438	14.068	21.471
Lysine	1.067	1.148	1.142
Methionine	0.298	0.301	0.300
Calcium	0.477	0.558	0.636
Sodium	0.218	0.218	0.220
Total phosphorus	0.574	0.582	0.617
Metabolizable energy, MJ/kg	11.412	11.401	11.102

CPH, cocoa pod husk; TvTD, *Talaromyces veruculosus* theobromine-degrading isolate.

^a^Inactivated TvTD-treated CPH or TvTD-treated CPH.

### Preparation of cocoa pod husk

Fresh husks of *Theobroma cacao* (cocoa) pods were collected from the Cocoa Research Institute of Ghana (CRIG), New Tafo-Akim, Eastern Region, Ghana. Cocoa pod husks were dried in an indirect solar dryer until moisture content was 9.8 ± 1.2%. The dried husks were milled to approximately 2 mm particle size and sterilized (121 °C and 15 psi for 20 min). Sterilization was to ensure that CPH substrate would be colonized only by the inoculum for biodetheobromination. Milled, sterilized CPH is subsequently referred to as substrate or untreated CPH.

### Reagents and chemicals

Kits for assays of enzyme activities, albumin, creatinine, total and direct bilirubin were procured from Cypress Diagnostics (Langdorp, Belgium). Theobromine, as well as reagents and chemicals for histopathological tissue processing, were procured from Sigma-Aldrich (St. Louis, MO).

### Bio-tool for detheobromination of cocoa pod husk

Detheobromination of CPH was by treatment with *Talaromyces verruculosus* KY697103 (TvTD), a filamentous fungus previously shown to be capable of theobromine-degradation^15^ and potentially useful as a bio-tool for detheobromination of CPH for animal feed^16^.

### Bio-detheobromination of cocoa pod husk

#### Treatment of cocoa pod husk

Spore suspensions of TvTD were prepared in sterile distilled water^19^ from 14-day-old cultures on theobromine-sucrose agar slants at room temperature (25–29 °C)^15^. Detheobromination of CPH was done using 1 kg of milled CPH per batch of treatment. Fungal seed culture for treatment was prepared in 200 mL theobromine liquid medium (TLM) inoculated to contain of 2 × 10^4^ spores/mL, and incubated with agitation at 90 strokes/min for 24 h at room temperature. Theobromine liquid medium^15^ was constituted with 0.2 mg/mL sucrose and 0.01 mg/mL theobromine. Theobromine was absent from control TLM. After the incubation period, seed cultures in control TLM were autoclaved to inactivate the inoculum^15^. Each seed culture was topped-up with distilled water to make 3 L of suspension and the entire volume was evenly mixed with 1 kg untreated CPH. Treatment vessels were loosely covered and left to stand for 10 days at room temperature.

#### Determination of theobromine content in cocoa pod husk

After the treatment period, CPH samples were autoclaved and dried to constant weight, for up to 5 days in an oven at 50 °C. Dried CPH, untreated or treated, was analyzed for theobromine content by a modification of the AOAC method for extraction of theobromine from cocoa beans^20^. Briefly, approximately 5.0 g of milled CPH were defatted with two portions of 30 mL petroleum ether (BDH, Poole, England) at room temperature, and left overnight in a fume hood to dry. The dried material was boiled in 100 mL distilled water for 20 min. After the period, an extra 50 mL distilled water was added twice, each time followed by boiling for 20 min. Between 70–90 mL of supernatant from each sample were concentrated by rotary evaporation under reduced pressure at 50 °C, filtered through a Whatman Spartan syringe filter with a 0.45 µm membrane (Sigma Aldrich; St. Louis, MO, USA) and analyzed for theobromine by high performance liquid chromatography (HPLC).

Chromatographic separation was done using a 20-µl sample volume at 27 °C on an Atlantis dC18 5 µm column (150 mm × 4.6 mm) fitted to a Waters HPLC unit consisting of a Waters In-Line degasser AF, Waters 1525 binary HPLC pump and Waters 2487 dual wavelength absorbance detector at 270 nm. Elution was by a solvent system of acetonitrile (ACN) and 0.1% formic acid adjusted to pH 3.75 with ammonia (AF), pumped for 20 min according to a gradient profile, with ACN:AF ratios starting from 2:98 through 12:88 and ending with 2:98^16^. Calibration plots were prepared from dilutions of a 0.1 mg/mL solution of theobromine. Internal theobromine controls were used to check for loss of theobromine during extraction from CPH. Also, analytical samples were spiked with theobromine for HPLC analysis, to confirm identity of the theobromine peak and also to validate quantitative deductions that were made from calibration plots.

### Feed formulations

Experimental feeds were formulated either with maize (control diet) or CPH replacing maize (substitution diets). Feeds were in the form of coarse powder. For the substitution diets, TvTD-treated or inactivated TvTD-treated CPH was included at 30% or 50% of the total diet (Table 1). Nutrient values for CPH used in feed formulations were based on values obtained from proximate analyses of untreated and TvTD-treated CPH^16^. Control and experimental diets were formulated to be isonitrogenous and isocaloric, estimated by calculation (Table 1).

### Animal selection and treatment

A total of 48 male Sprague–Dawley rats (180–255 g) were selected for the study. For mating, 18 non-pregnant, nulliparous, female Sprague–Dawley rats (160–180 g) were selected. The male rats were assigned to 6 feeding groups of 8 animals each. After acclimatization, baseline values for body weight, full blood count and blood biochemistry were recorded. The designations and treatment regimen of the 6 feeding groups were as follows: Group 1 (CF), control diet (regular rat chow); Group 2 (CT), control diet + theobromine (300 mg/kg BW); Group 3 (ITC-30), base diet substituted with 30% inactivated TvTD-treated CPH; Group 4 (TC-30), base diet substituted with 30% TvTD-treated CPH; Group 5 (ITC-50), base diet substituted with 50% inactivated TvTD-treated CPH; Group 6 (TC-50), base diet substituted with 50% TvTD-treated CPH. Excess feed and drinking water were available to the rats for a period of 12 weeks.

#### Mortality

Throughout the 12-week period, mortality of any animal in the treatment groups was recorded. Only surviving animals were used as statistical units for subsequent measurements and analyses.

#### Water and feed intake

Three rats from each feeding group were housed individually in metabolic cages and had free access to measured excesses of feed and water daily for 12 weeks. Quantities of feed and water consumed over the first 7 days after the start of animal feeding were used to establish baseline values. The weekly changes in feed and water intakes for each feeding group were expressed as percentages of their respective baseline values. Total feed and water intakes over the period were determined by area under curve (AUC) analysis of the mean weekly intake values for each feeding group.

#### Body weight

Body weight of all the animals in each feeding group was determined before the start of feeding and then weekly thereafter over the experimental period. Total percentage changes in body weight were determined by AUC analysis of the mean weekly values for each feeding group.

### Hematological analysis

All rats were sampled for baseline hematology values. Subsequently, the rats were sampled for blood at 4-week intervals from start until termination of feeding trial. Blood, approximately 1 mL, for hematological analyses was drawn by tail snip, collected into EDTA-coated tubes and analyzed within 24 h. Full blood counts were measured from the uncoagulated blood samples using a Sysmex KX-21N Automated Haema Screen (Ontario, Canada).

### Biochemical analyses

Blood for biochemical analyses (3 mL) was drawn from all rats into tubes on ice, allowed to clot and centrifuged at 4,000×*g* and 10 °C for 10 min. The supernatant serum was collected and used for analyses. For storage, samples were kept at − 20 °C. Biochemical analyses for heart, liver and kidney functions were conducted. The marker used for assessing cardiac function was creatine kinase MB (CK-MB) isoenzyme. For assessing liver function, albumin, total and direct bilirubin concentrations were determined. Creatinine levels were measured for kidney function.

### Mating

At termination of feeding trial, 3 male rats from each feeding group were introduced to 3 female rats each. Number of animals used for mating was limited by mortalities in some treatment groups. After allowing 5 days for mating, the males were removed, weighed and sacrificed. Testes and thymus were excised, weighed and processed for histological analyses. Females were observed throughout parturition and upon delivery of pups, litter characteristics were recorded.

### Organ wet weights

After the feeding trial and mating, 5 animals from each feeding group (3 animals from the CT group) were sacrificed by cervical dislocation. Selected organs (thymus, liver, kidney, heart, spleen, lungs and testes) were excised, blotted dry and weighed to investigate the effect of the different feeds on mean organ wet weight expressed as a percentage of body weight.

### Histology

Histology of excised organs (liver, testes and thymus) was carried out in accordance with established protocol at the Electron Microscopy Unit of Noguchi Memorial Institute for Medical Research, University of Ghana, and as described in the Manual of Histological Staining Methods^21^ using hematoxylin and eosin. Stained tissues were observed under a light microscope at 100 × or 200 × magnification, and pictures were taken by a camera attached to an Olympus BH-2 photomicroscope (New York Microscope Co., Hicksville, NY, USA).

### Statistical analysis

All statistical analyses were performed with data analyses tools in Microsoft Excel version 14, Microsoft Office Professional Plus 2010. For determining significance of differences, data were analyzed by one-way ANOVA and post hoc by Tukey’s multiple comparison test on GraphPad Prism version 8. Analysis of trends for changes in growth parameters was by EpiTools Epidemiological Calculators^22^. All analyses were conducted at *α* = 0.05. Individual rats were used as statistical units for analyses. Except for the CT group which had *n* = 3 for statistical analyses, all other groups had *n* ≥ 5 unless otherwise indicated.

## Results

### Bio-detheobromination of cocoa pod husk

**Figure 1:**
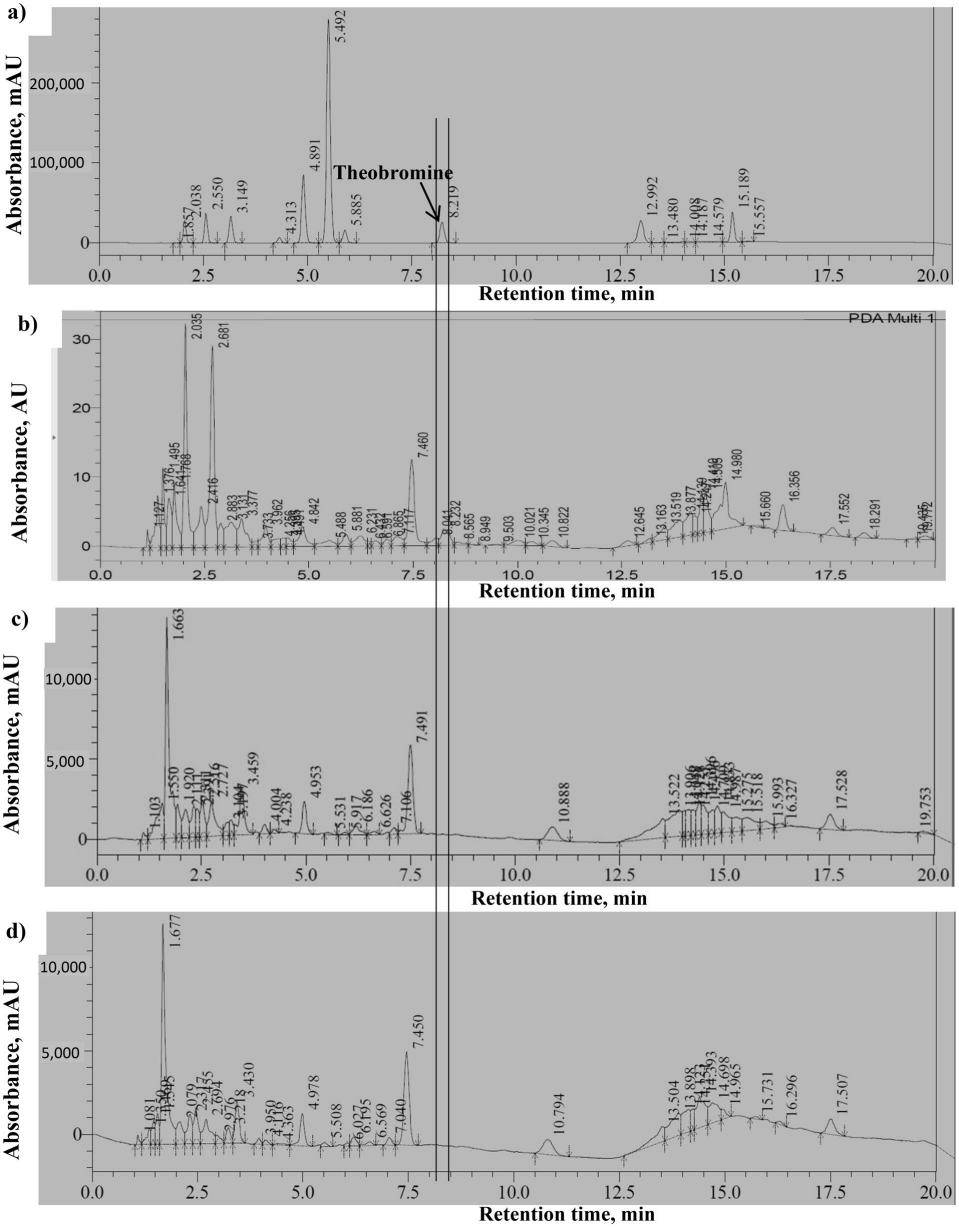
High performance liquid chromatography output for (**A**) a mixture of methylxanthines, and aqueous extracts of (**B**) control, (**C**) sterilized, TvTD-treated and (**D**) unsterilized, TvTD-treated CPH. TvTD, *Talaromyces verruculosus*; CPH, cocoa pod husk. A vertical line through all the plots indicates the expected elution time for theobromine. CPH was treated with TvTD for 10 days. The aqueous extracts were scanned for all absorbing compounds in the range 190–400 nm.

HPLC analysis of aqueous extracts of CPH samples indicated that unlike inactivated TvTD-treated CPH which had a peak for theobromine at retention time of 8.232 min, peaks for theobromine were not detected in sterilized or unsterilized TvTD-treated samples (Fig. 1). Limit of detection of theobromine in CPH was approximately 0.07 mg/g. In theobromine extracts/solutions, the lowest concentrations detected or quantified were approximately 4 µg/mL and 15.0 µg/mL, respectively.

By calculation, approximate values for total energy and total nitrogen contents of diets used in the study were 11.3 MJ/kg and 17.6%, respectively (Table 1).

### Mortality

**Figure 2:**
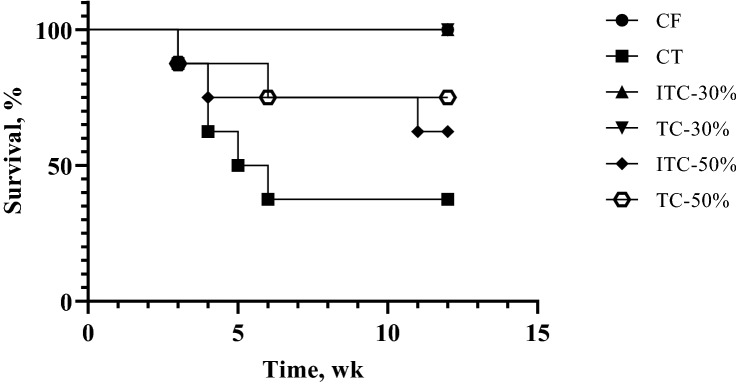
Kaplan–Meier survival curve representing mortalities recorded among rats in each treatment group. TvTD, *Talaromyces verruculosus* theobromine-degrading isolate; CPH, cocoa pod husk. Number of animals in each group = 8. Diet formulations were either regular rat feed or rat feed with TvTD-treated or untreated (control) CPH substituting maize at 30% or 50%, respectively.

Of 8 Sprague–Dawley rats in each feeding group, mortalities recorded over the 12-week period are as shown in Fig. 2. For the control group on base diet (CF), 30% TvTD-treated (TC-30) and 30% inactivated TvTD-treated (ITC-30) CPH substitution groups, no animals died during the period of the study. In contrast, five rats died over the 12-week period in the control group on base diet + theobromine (CT), whereas three and two rats respectively died in the 50% TvTD-treated (TC-50) and 50% inactivated TvTD-treated (ITC-50) CPH substitution groups.

### Feed and water intake

**Figure 3:**
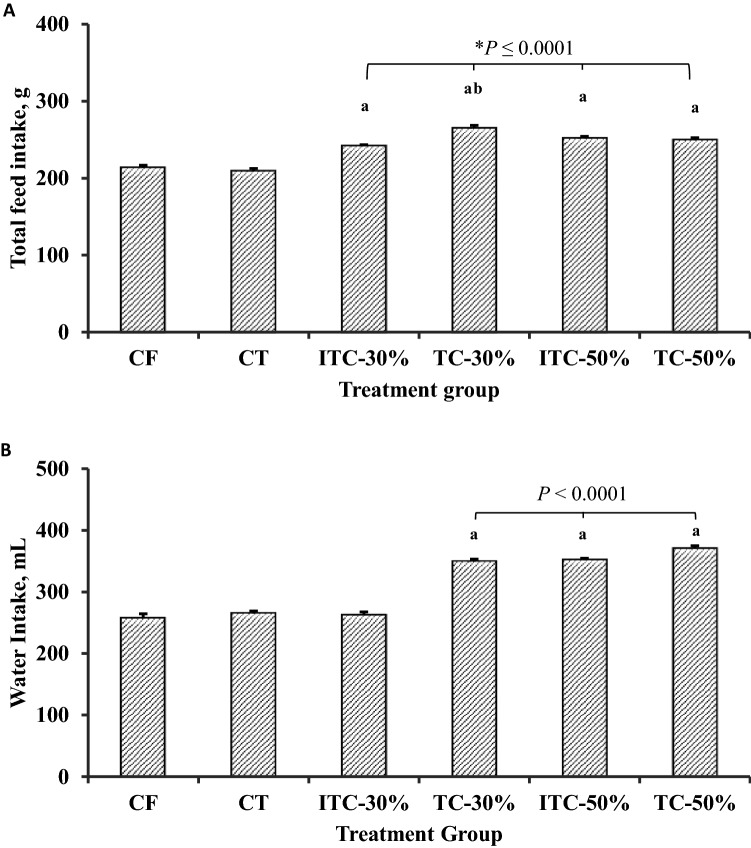
Effect of various feeds on (**A**) total feed intake and (**B**) total water intake by Sprague–Dawley rats at termination of feeding trial. TvTD, *Talaromyces verruculosus*; CPH, cocoa pod husk; CF, control feed; CT, control feed with theobromine; ITC-30%, 30% inactivated TvTD-treated CPH substituted feed; TC-30%, 30% TvTD-treated CPH substituted feed; ITC-50%, 50% inactivated TvTD-treated CPH substituted feed; TC-50%, 50% TvTD-treated CPH substituted feed. Each point represents mean ± SEM of *n* ≥ 3. ^a^Significantly higher than CF value (*P* < 0.05). ^b^Significantly higher than value for ITC-30% (*P* < 0.05).

There was no significant difference in total feed intake between the CF and CT feed groups (Fig. 3A). Relative to both control groups, total feed intake for all the CPH substitution feed groups was significantly higher (*P* < 0.0001). Whereas total intake for TC-30 was significantly higher than ITC-30 group (*P* = 0.0001), there was no significant difference in total feed intake between the 50% substitution groups. With reference to water intake, there were no significant differences between the CF, CT and ITC-30 groups, whereas all others had significantly higher (*P* < 0.0001) water intake values (Fig. 3B).

### Change in body weight

**Figure 4:**
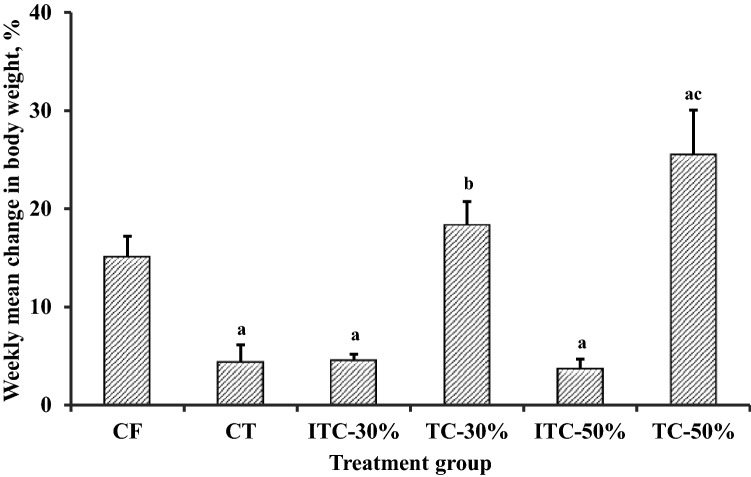
Effect of various feeds on changes in body weight of Sprague–Dawley rats at termination of feeding trial. TvTD, *Talaromyces verruculosus*; CPH, cocoa pod husk; CF, control feed; CT, control feed with theobromine; ITC-30%, 30% inactivated TvTD-treated CPH substituted feed; TC-30%, 30% TvTD-treated CPH substituted feed; ITC-50%, 50% inactivated TvTD-treated CPH substituted feed; TC-50%, 50% TvTD-treated CPH substituted feed. Body weights were measured weekly and changes were expressed as percentages of the mean baseline body weight for each group. Each bar represents mean ± SEM of *n* ≥ 3 of total percentage weight change as area under time-course curve (not shown). ^a^Significantly different from CF value (*P* < 0.05). ^b^Significantly higher than value for ITC-30% (*P* < 0.05). ^c^Significantly higher than values for TC-30% and ITC-50% (*P* < 0.05).

Increases in body weight relative to baseline values were recorded for all feeding groups (Table 2; Fig. 4). Chi-squared test for trends however indicated that the positive slopes of body weight changes for ITC-30 and ITC-50 were not significantly different (*P* ≥ 0.5144) from zero (Table 2). Weight gain by CT, ITC-30 and ITC-50 were significantly less than the value for CF (*P* ≤ 0.035) (Fig. 4). Weight gain for TC-30 was not different from CF whereas weight gain for TC-50 was significantly higher (*P* = 0.0394).

**Table 2:** Trend analysis of mean percentage changes in animal body weight.

Feeding group	Approximate weight gain from baseline, %	Slope	*P*-value	Comment
CF	22	0.00397	0.0057	Positive, non-zero slope
CT	9	0.00309	0.0397	Positive, non-zero slope
ITC-30%	4	0.00084	0.5899	Positive, not significantly different from zero
TC-30%	22	0.00388	0.0201	Positive, non-zero slope
ITC-50%	6	0.00127	0.5144	Positive, not significantly different from zero
TC-50%	47	0.00783	0.0001	Positive, non-zero slope

CF, control feed; CT, control feed with theobromine; TvTD, *Talaromyces verruculosus*; ITC-30%, 30% inactivated TvTD-treated cocoa pod husk (CPH) substituted feed; TC-30%, 30% TvTD-treated CPH substituted feed; ITC-50%, 50% inactivated TvTD-treated CPH substituted feed; TC-50%, 50% TvTD-treated CPH substituted feed.

Significance of slope of the trend line for each feeding group was analysed by Chi-squared test for trends with *α* = 0.05.

### Hematology

No differences were observed between the CF and CT groups. Relative to CF, white blood cell (WBC) counts of ITC-30 and TC-50 were significantly (*P* ≤ 0.0275) different (Table 3). Except for ITC-30, animals in CPH substituted feed groups had mean cell hemoglobin (MCH) significantly (*P* ≥ 0.0348) lower than the CF value. Animals in all CPH substituted groups had mean cell volume (MCV) significantly (*P* ≥ 0.0091) lower than CF. Both ITC groups recorded significantly (*P* ≥ 0.0286) lowered RBC counts, by at least 15%. All CPH substitution groups, except TC-30, recorded significantly (*P* ≥ 0.0297) reduced hemoglobin (HGB) and hematocrit (HCT). However, none of the feeds affected mean cell hemoglobin concentration (MCHC) platelet (PLT) counts and red blood cell distribution width (RDW).

**Table 3:** Effects of feed on hematological indices of rats at termination of feeding^1^.

Feeding group	WBC, × 10^3^ µL	RBC, × 10^6^ µL	HGB, × g/dL	HCT, %	MCV, fL	MCH, pg	MCHC, g/dL	PLT, × 10^3^ µL	RDW_SD, fL	MPV, fL
CF	17.33 ± 4.46	9.28 ± 0.42	16.96 ± 0.75	52.33 ± 2.14	58.66 ± 0.43	18.83 ± 0.28	31.56 ± 0.31	980.66 ± 87.87	35.23 ± 1.47	7.56 ± 0.08
CT	11.43 ± 0.17	8.45 ± 0.17	15.56 ± 0.20	50.03 ± 0.54	59.73 ± 0.38	18.16 ± 0.31	32.16 ± 0.61	945.33 ± 43.10	31.36 ± 0.50	7.1 ± 0.06
ITC-30%	^a^7.26 ± 1.16	^a^7.88 ± 0.07	^a^13.7 ± 0.21	^a^43.7 ± 0.31	^a^55.43 ± 0.32	17.4 ± 0.32	31.36 ± 0.31	966.66 ± 94.84	28.03 ± 0.20	6.83 ± 0.14
TC-30%	10.70 ± 3.82	8.9 ± 0.34	15.16 ± 0.56	48.23 ± 1.75	^a^54.23 ± 0.44	^a^17.06 ± 0.07	31.43 ± 0.32	1,069.33 ± 42.27	26.8 ± 0.12	6.83 ± 0.08
ITC-50%	10.43 ± 0.35	^a^7.78 ± 0.09	^a^12.96 ± 0.21	^a^44.56 ± 1.26	^a^55.5 ± 0.7	^a^17.23 ± 0.20	32.16 ± 0.69	976.36 ± 5.35	28.86 ± 0.21	6.83 ± 0.06
TC-50%	^a^ 8.3 ± 2.16	8.50 ± 0.29	^a^13.4 ± 0.88	^a^43.3 ± 1.93	^a^50.86 ± 0.67	^a,b^15.7 ± 0.53	30.9 ± 0.75	1,222.67 ± 135.17	29.00 ± 0.70	6.46 ± 0.08

WBC, white blood cells; RBC, red blood cells; HGB, haemoglobin; HCT, haematocrit; MCV, mean corpuscular volume; MCH, mean cell haemoglobin; MCHC, mean cell haemoglobin concentration; PLT, platelets; RDW_SD, red blood cell distribution width; MPV, mean platelet volume; CF, control feed; CT, control feed with theobromine; CPH, cocoa pod husk; TvTD, *Talaromyces verruculosus*; ITC-30%, 30% inactivated TvTD-treated CPH substituted feed; TC-30%, 30% TvTD-treated treated CPH substituted feed; ITC-50%, 50% inactivated TvTD-treated CPH substituted feed; TC-50%, 50% TvTD-treated CPH substituted feed.

^a^Significantly lower than CF value (*P* < 0.05).

^b^Significantly lower than ITC-50% value (*P* < 0.05).

^1^Sprague–Dawley rats were fed with the indicated formulations over a feeding trial period of 12 weeks. Values shown are means ± SEM for *n* ≥ 3.

### Mean organ wet weights

**Figure 5:**
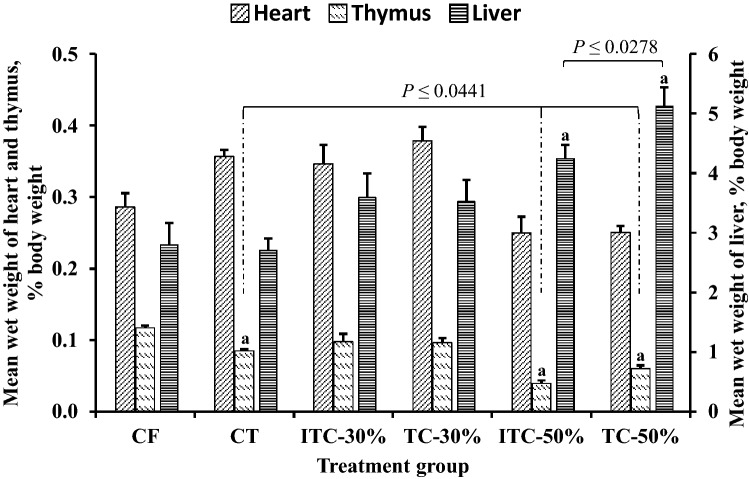
Effect of various feeds on selected organ wet weights of Sprague–Dawley rats at termination of feeding trial. TvTD, *Talaromyces verruculosus*; CPH, cocoa pod husk; CF, control feed; CT, control feed with theobromine; ITC-30%, 30% inactivated TvTD-treated CPH substituted feed; TC-30%, 30% TvTD-treated CPH substituted feed; ITC-50%, 50% inactivated TvTD-treated CPH substituted feed; TC-50%, 50% TvTD-treated CPH substituted feed. Wet weight of each organ was expressed as a percentage of body weight of animal. Each bar represents mean ± SEM for *n* ≥ 3. ^a^Significantly different from corresponding organ control (*P* < 0.05).

Substitution with CPH at 50% significantly increased mean liver wet weight by 51% to 83% (*P* ≤ 0.0278), relative to CF (Fig. 5). No differences between control and treatment groups were observed for mean heart wet weight. Compared to the control group, mean wet weights of thymus in animals from CT and 50% CPH substituted groups were significantly lower (*P* ≤ 0.0441) by 13–66% (Fig. 5).

### Serum biochemistry

**Figure 6:**
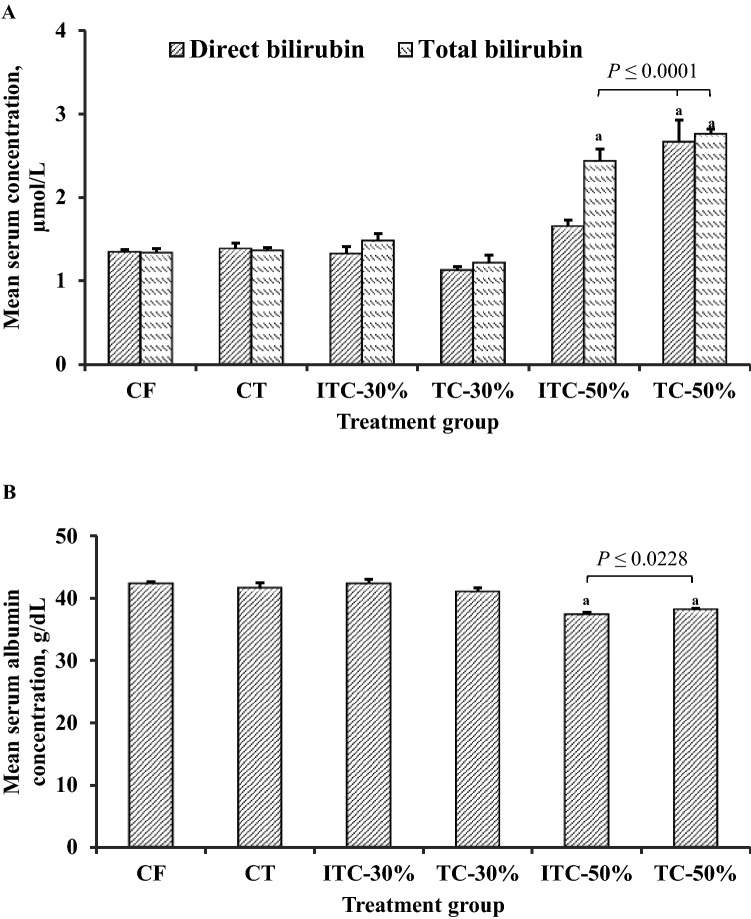
Effect of various feeds on indicators of liver function and integrity; (**A**) direct or total bilirubin and (**B**) serum albumin concentrations in Sprague–Dawley rats at termination of feeding trial. TvTD, *Talaromyces verruculosus*; CPH, cocoa pod husk; CF, control feed; CT, control feed with theobromine; ITC-30%, 30% inactivated TvTD-treated CPH substituted feed; TC-30%, 30% TvTD-treated CPH substituted feed; ITC-50%, 50% inactivated TvTD-treated CPH substituted feed; TC-50%, 50% TvTD-treated CPH substituted feed. Each bar represents mean ± SEM of *n* ≥ 3. ^a^Significantly different from CF value (*P* < 0.05).

**Figure 7:**
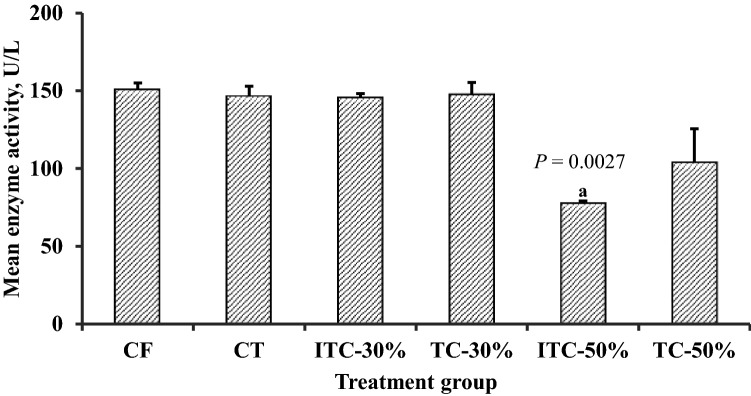
Effect of feed formulations on serum creatine kinase-MB activity in Sprague–Dawley rats at termination of feeding trial. TvTD, *Talaromyces verruculosus*; CPH, cocoa pod husk; CF, control feed; CT, control feed with theobromine; ITC-30%, 30% inactivated TvTD-treated CPH substituted feed; TC-30%, 30% TvTD-treated CPH substituted feed; ITC-50%, 50% inactivated TvTD-treated CPH substituted feed; TC-50%, 50% TvTD-treated CPH substituted feed. Each bar represents mean ± SEM of *n* ≥ 3. ^a^Significantly different from CF value (*P* < 0.05).

To further evaluate organ-directed effects of theobromine or CPH substituted feeds on the kidneys, liver and heart, selected serum biochemical indices were measured. Assessment of kidney function by serum creatinine concentration showed no significant differences between the control and any other feeding group (data not shown). For liver function, the 50% CPH substitution groups showed significantly higher total bilirubin concentration (*P* < 0.0001) (Fig. 6A). Direct bilirubin was high only for TC-50 group (*P* < 0.0001) (Fig. 6B). Again, the 50% CPH substitution groups had significantly lower (≥ 9%) serum albumin concentrations (*P* ≤ 0.0228) (Fig. 6B). Assessment of cardiac function showed significant reductions (≥ 35%) in CK-MB activity in animals from the ITC-50 group (*P* = 0.0057) (Fig. 7).

### Litter characteristics

**Figure 8:**
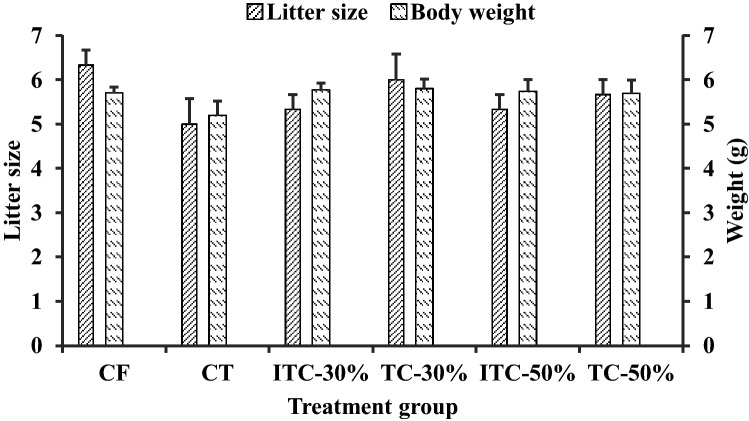
Litter size and body weight at birth of offspring of male Sprague–Dawley rats used in feeding trial. CF, control feed; CT, control feed with theobromine; ITC-30%, 30% inactivated TvTD-treated CPH substituted feed; TC-30%, 30% TvTD-treated CPH substituted feed; ITC-50%, 50% inactivated TvTD-treated CPH substituted feed; TC-50%, 50% TvTD-treated CPH substituted feed. Each bar represents mean ± SEM of *n* = 3.

Relative to CF, there were no overt effects of theobromine administration or CPH substituted feeds on litter size or birth weight (Fig. 8). No deformities or other signs of toxicity were observed in the rat pups.

### Histopathology

**Figure 9:**
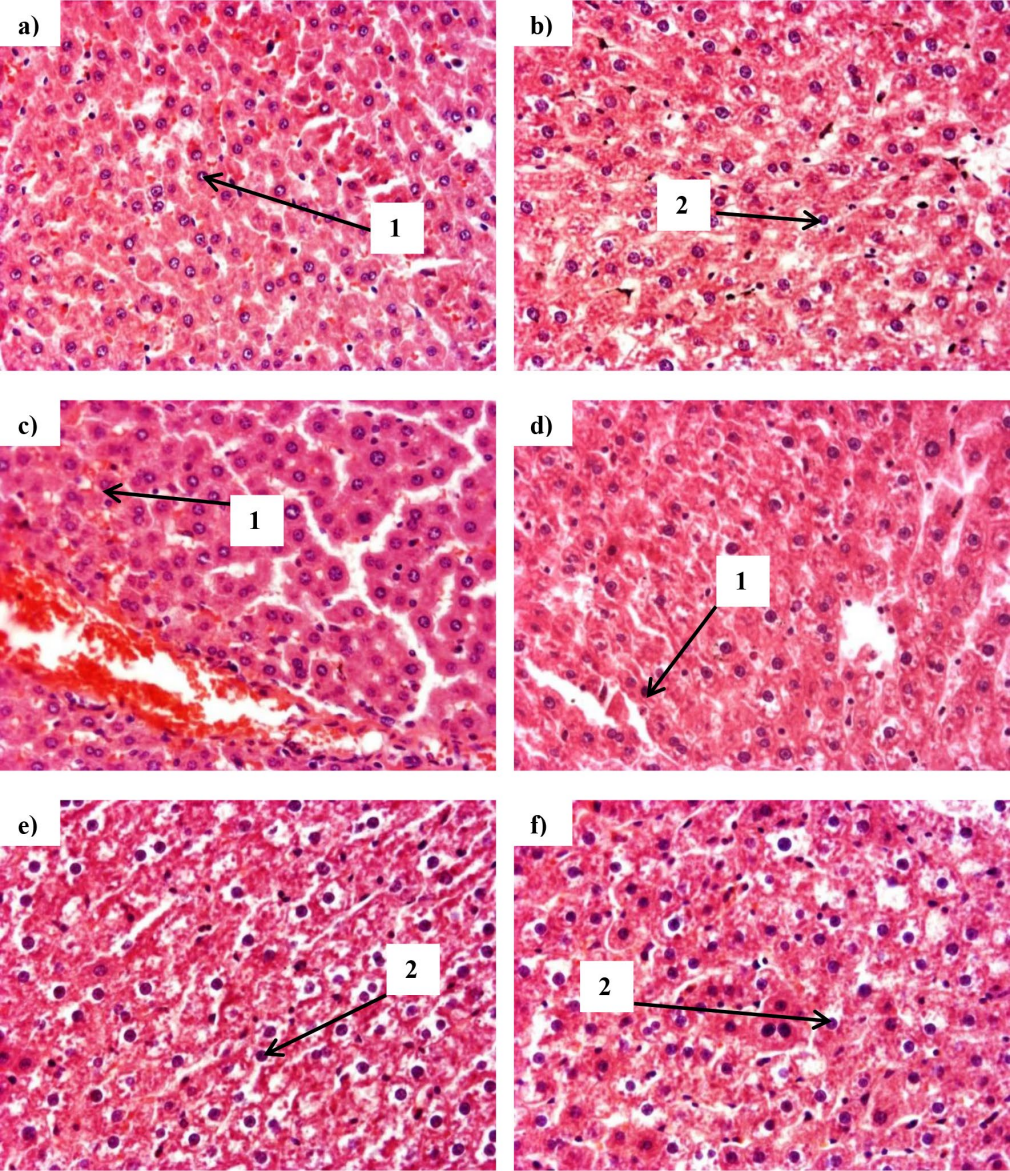
Representative micrographs of liver tissue at termination of animal feeding trial. TvTD, *Talaromyces verruculosus*; CPH, cocoa pod husk. The sections show normal liver cells for (**A**) control feed, (**B**) control feed with theobromine, (**C**) 30% inactivated TvTD-treated CPH substituted feed, (**D**) 30% TvTD-treated CPH substituted feed, (**E**) 50% inactivated TvTD-treated CPH substituted feed and (**F**) 50% TvTD-treated CPH substituted feed. Cells appear normal (1) in (**A**), (**C**) and (**D**) but necrotic (2) in (**B**), (**E**) and (**F**). Magnification: × 200.

Micrographs of liver sections showed that theobromine administration and 30% CPH-substituted feeds (TC-30 and ITC-30) had no effects on liver cells (Fig. 9B–D) whereas 50% substitution with CPH (TC-50 and ITC-50) caused necrosis of liver cells (Fig. 9E,F).

**Figure 10:**
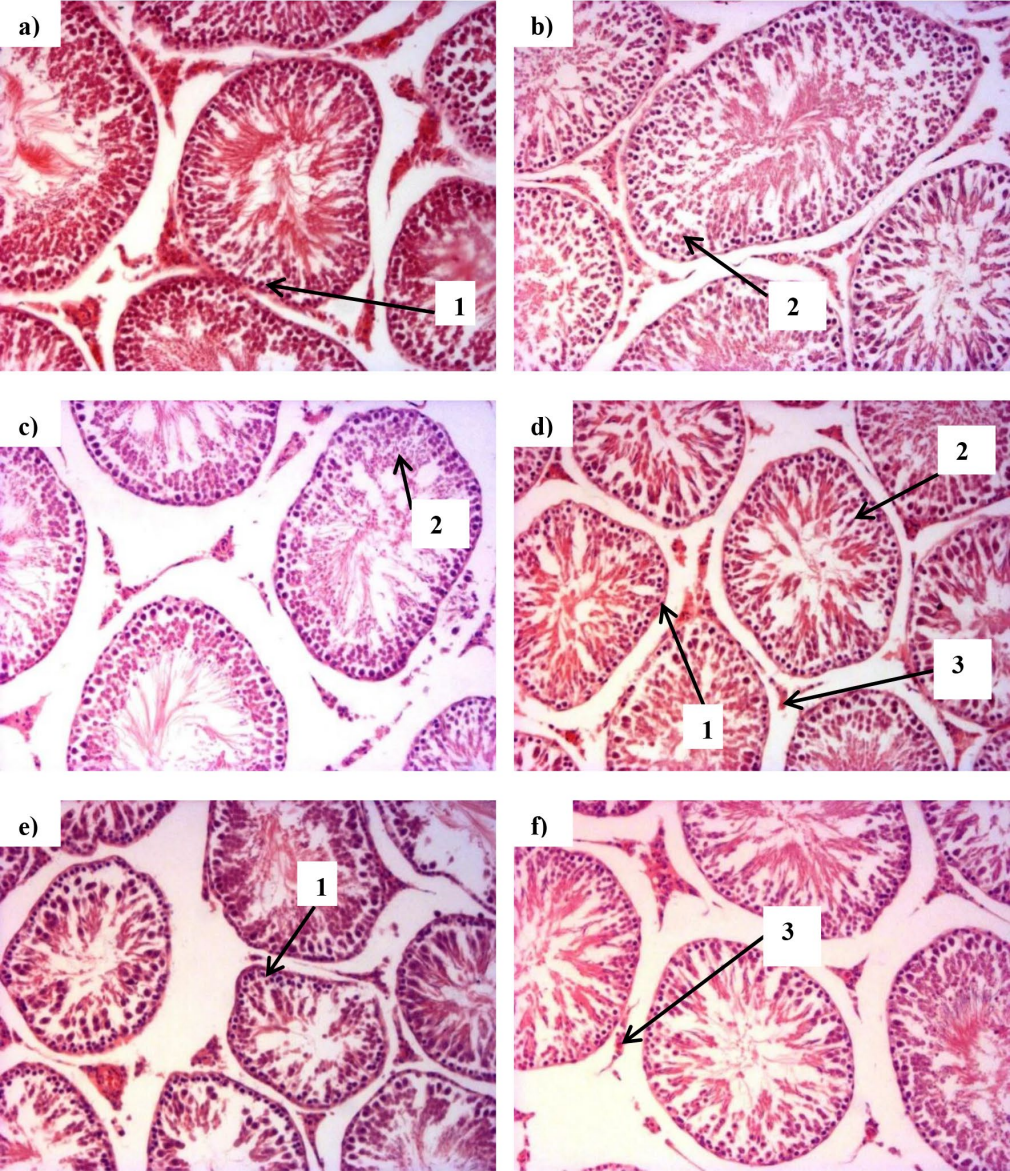
Representative micrographs of testes tissue at termination of animal feeding trial. TvTD, *Talaromyces verruculosus*; CPH, cocoa pod husk. The sections show spermatogonia (1), spermatids (2) and leydig cells (3) for (**A**) control feed, (**B**) control feed with theobromine, (**C**) 30% inactivated TvTD-treated CPH substituted feed, (**D**) 30% TvTD-treated CPH substituted feed, (**E**) 50% inactivated TvTD-treated CPH substituted feed and (**F**) 50% TvTD-treated CPH substituted feed. Magnification: × 100.

Micrographs of testes of animals from all groups, except CT, showed normal-shaped cells with spermatogonia and spermatids (Fig. 10A,C–F). On the other hand, the CT group showed elongated cells (Fig. 10B).

**Figure 11:**
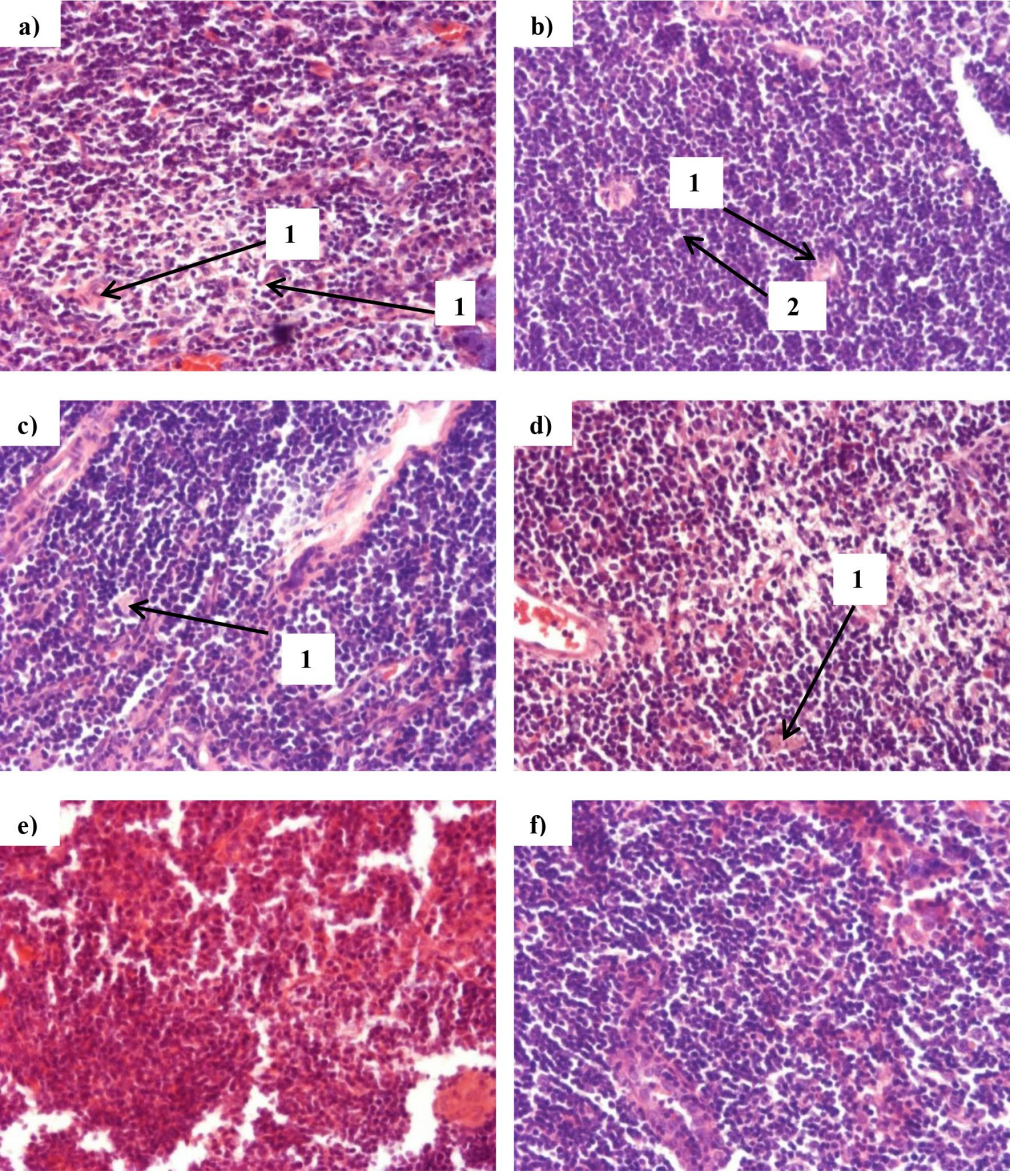
Representative micrographs of thymic tissue at termination of animal feeding trial. TvTD, *Talaromyces verruculosus*; CPH, cocoa pod husk. Thymus sections with visible Hassal’s corpuscles (1) are shown for (**A**) control feed, (**B**) control feed with theobromine, (**C**) 30% inactivated TvTD-treated CPH substituted feed, (**D**) 30% TvTD-treated CPH substituted feed, (**E**) 50% inactivated TvTD-treated CPH substituted feed and (**F**) 50% TvTD-treated CPH substituted feed. Extensive disorganization and/or damage are seen in (**E**). Magnification: × 200.

Micrographs of the thymus revealed tissue disorganization in ITC-50 animals (Fig. 11E). In all other groups except CF and TC-30, indications of tissue striation were observed (Fig. 11A–D,F).

## Discussion

Inclusion of CPH in animal feed formulations has been limited mainly due to its constituent compounds, principally theobromine^4,12,23^. To investigate nutritional value and safety of bio-detheobrominated CPH, TvTD-treated CPH at dietary substitution levels of 30% and 50% were tested in male Sprague–Dawley rats. As positive control, theobromine was administered to a group of the animals on control diet. Preliminary experiments conducted as part of this study indicated that a daily theobromine dose of 500 mg/kg BW^24^ was extremely toxic, with ≥ 80% of Sprague–Dawley rats dying within 2 weeks of administration. A similar observation has previously been made^25^. The dose of theobromine for this study was, therefore, reduced to 300 mg/kg BW. Inactivated TvTD-treated CPH contained 1.51 ± 0.06 mg theobromine per g of husks.

For the scaled-up treatment of CPH, a seed culture was used, as opposed to a spore suspension, because it was desired to induce the theobromine-degrading bio system of the isolate^15^ to ensure maximum possible detheobromination. No differences were observed in HPLC chromatograms of sterilized and unsterilized TvTD-treated CPH. This suggested that with reference to chemical composition detectable by HPLC conditions used in the study, exposure to heat during sterilization by autoclaving may not have had any effect on TvTD-treated CPH. Diets used in this study were formulated to be isonitrogenous and isocaloric by adjusting the content of various components in feed formulations (Table 1).

Feed with CPH substitution at 30% or 50% resulted in higher mean feed and water intake values relative to the normal control feed (Fig. 2). This may be attributable to the higher fiber content of the CPH-substituted feeds relative to the control feed. Rats adapt to high fiber diets^26^, and may also adapt to low nutrient diets, by increasing feed intake whilst apparent digestibility may be reduced. Also, because transit time of food through the gastrointestinal tract generally decreases with increasing fiber content^26,27^, increases in frequency of feeding along with corresponding increases in water intake were expected with increasing CPH substitution in feed.

Increases in feed and water intakes by the animals on CPH-substituted feeds did not always result in corresponding increases in body weight (Fig. 3), suggesting that weight gain was not a direct reflection of feed or water intake. Control feed with theobromine or feed substitution with inactivated TvTD-treated CPH significantly reduced mean body weight of animals. Because diets used for the different feeding groups were formulated to be isonitrogenous and isocaloric, this observation strongly confirms the detrimental effect of theobromine on feed conversion, and consequently on weight gain^25^. This effect was not observed with the diets containing CPH that had been detheobrominated by TvTD treatment.

Theobromine administration resulted in a decrease in thymus wet weight but had no such effect on the liver. Also, animals on feeds with 50% CPH substitution showed marked increases or decreases in liver or thymus wet weights, respectively, suggesting possible adverse effects not attributable only to theobromine. The extent of atrophy of the thymus in animals on 50% CPH substituted feeds, albeit to a lesser degree in the TvTD-treated CPH substituted feed, correlates well with the adverse morphological effects observed in the thymus for these two feeding groups (Fig. 11). This difference observed between the 50% CPH substitution feeds may have been due to the presence of theobromine in the inactivated TvTD-treated CPH feed. Based on micrographs for the treatment groups, only animals on 30% TvTD-treated CPH substitution showed no indications of cellular damage to the thymus.

Theobromine is known to have pronounced adverse effects on the testes in male rats^9,10,24^. Micrographs of the testes showed normal-shaped cells for all feeding groups except for the group on control feed with theobromine. The micrograph for this group showed cells that had assumed elongated oval shapes, contrary to the relatively spherical shapes observed for all others. This abnormal testicular morphology is, however, unlikely to have caused problems with organ integrity and/or osmoregulation since it did not affect the mean wet weight of the testis relative to the normal control. The levels of theobromine in the inactivated TvTD-treated CPH substituted feeds may have been too low to affect cell morphology. All the male animals used in the breeding experiment successfully mated and impregnated their assigned female counterparts. After delivery of pups, no abnormal litter characteristics or teratogenic effects were observed relative to the control, suggesting further that there were no adverse reproductive or genotoxic effects expressed by the feeds.

Serum biochemistry indices showed significant increases in total and direct serum bilirubin levels and reductions in serum albumin concentration in the animals on 50% CPH-substituted feeds. Liver damage is known to impair excretory and synthetic liver functions. This may also affect osmoregulation in hepatocytes and lead to edema of the liver^28^, hence the increased mean liver wet weights recorded for animals on 50% substituted diets. These findings are corroborated by the morphology of liver tissue of animals fed on 50% CPH-substituted feeds, which showed necrosis of liver cells.

CPH substitution, particularly that of the inactivated TvTD-treated CPH, reduced most of the indices; suggesting that the composition of CPH directly affected the blood cells and/or hematopoietic processes^29,30^. The adverse effects on RBC, WBC, MCV and hemoglobin concentration, at both levels of CPH feed substitution, albeit to a higher degree at 50% substitution is of concern since this may cause anemia, reduce immune defense and affect general well-being of the animals. Regular feed with theobromine had considerable adverse effects on WBC and MPV, also an indication of probable negative impact on the immune system. However, the role played by theobromine is unclear because reduction in theobromine levels of CPH, as a result of TvTD treatment, is not reflected in reduced adverse hematological effects of the substituted feed formulations.

Based on the promising findings from this study, efforts are underway for collaboration and funding to conduct a similar feeding study using poultry, and subsequently sheep and pigs. This bioremediation approach is easily applicable to other substrates whose use in animal feed is hindered by considerable methylxanthine content, such as coffee husks^16^.

## Conclusions

Using TvTD-treated CPH in place of maize, at a substitution level of 30% of feed, did not impact negatively on growth performance, physiology and reproduction of male Sprague–Dawley rats. Relative to regular feed (control), feed substitution at 30% with TvTD-treated CPH was comparable with respect to weight gain of the rats, albeit advantageous at 50% substitution. However, the observed indications of microcytic anemia due to CPH substituted diets is of concern and may be addressed, possibly by use of micronutrients.
